# A co-ultramicronized palmitoylethanolamide/luteolin composite mitigates clinical score and disease-relevant molecular markers in a mouse model of experimental autoimmune encephalomyelitis

**DOI:** 10.1186/s12974-019-1514-4

**Published:** 2019-06-20

**Authors:** Gabriella Contarini, Davide Franceschini, Laura Facci, Massimo Barbierato, Pietro Giusti, Morena Zusso

**Affiliations:** 1 Department of Pharmaceutical and Pharmacological Sciences, University of Padua, Largo Meneghetti, 2, 35131 Padua, Italy; 2 Present address: Selvita S.A. Park Life Science ul., Bobrzyńskiego, 14 30-348 Kraków, Poland

**Keywords:** Multiple sclerosis, EAE, Acute phase response, PEALut

## Abstract

**Background:**

Persistent and/or recurrent inflammatory processes are the main factor leading to multiple sclerosis (MS) lesions. The composite ultramicronized palmitoylethanolamide, an endogenous N-acylethanolamine, combined with the flavonoid luteolin, PEALut, have been found to exert neuroprotective activities in experimental models of spinal and brain injury and Alzheimer disease, as well as a clinical improvement in human stroke patients. Furthermore, PEALut enhances the expression of different myelin proteins in oligodendrocyte progenitor cells suggesting that this composite might have protective effects in MS experimental models.

**Methods:**

The mouse model of experimental autoimmune encephalomyelitis (EAE) based on active immunization with a fragment of myelin oligodendrocyte glycoprotein (MOG_35-55_) was used. The daily assessment of clinical score and the expression of serum amyloid A (SAA1), proinflammatory cytokines TNF-α, IL-1β, IFN-γ, and NLRP3 inflammasome, as well as TLR2, Fpr2, CD137, CD3-γ, and TCR-ζ chain, heterodimers that form T cell surface glycoprotein (TCR), and cannabinoid receptors CB_1_, CB_2_, and MBP, were evaluated in the brainstem and cerebellum at different postimmunization days (PIDs).

**Results:**

Vehicle-MOG_35-55_-immunized (MOG_35-55_) mice developed ascending paralysis which peaked several days later and persisted until the end of the experiment. PEALut, given intraperitoneally daily starting on day 11 post-immunization, dose-dependently improved clinical score over the range 0.1–5 mg/kg. The mRNA expression of SAA1, TNF-α, IL-1β, IFN-γ, and NLRP3 were significantly increased in MOG_35-55_ mice at 14 PID. In MOG_35-55_ mice treated with 5 mg /kg PEALut, the increase of SAA1, TNF- α, IL-1β, and IFN-γ transcripts at 14 PID was statistically downregulated as compared to vehicle-MOG_35-55_ mice (*p* < 0.05).

The expression of TLR2, Fpr2, CD137, CD3-γ, TCR-ζ chain, and CB_2_ receptors showed a significant upregulation in vehicle-MOG_35-55_ mice at 14 PID. Instead, CB_1_ and MBP transcripts have not changed in expression at any time. In MOG/PEALut-treated mice, TLR2, Fpr2, CD137, CD3-γ, TCR-ζ chain, and CB_2_ mRNAs were significantly downregulated as compared to vehicle MOG_35-55_ mice.

**Conclusions:**

The present results demonstrate that the intraperitoneal administration of the composite PEALut significantly reduces the development of clinical signs in the MOG_35-55_ model of EAE. The dose-dependent improvement of clinical score induced by PEALut was associated with a reduction in transcript expression of the acute-phase protein SAA1, TNF-α, IL-1β, IFN-γ, and NLRP3 proinflammatory proteins and TLR2, Fpr2, CD137, CD3-γ, TCR-ζ chain, and CB_2_ receptors.

## Background

Multiple sclerosis (MS) is one of the world’s most common neurologic disorders, and in many countries, it is the leading cause of non-traumatic neurologic disability in young adults [1]: it is estimated that MS affects approximately 2.3 million people worldwide [1, 2], with increased incidence in female [2]. Although widely believed to be immune-mediated and pathologically attributable to myelin-specific autoreactive CD4^+^ T cells, the humoral autoimmune response in MS is probably not restricted to myelin but affects the entire brain. The complex heterogeneity of MS is proven by the finding that auto-antibodies are formed against different CNS cell types, including neurons, oligodendrocytes, astrocytes, and immune cells [3]. Effector cells of both the innate and adaptive immune systems, e.g., microglia, activated macrophages, mast cells, and B and T lymphocytes, are all known to influence the pathogenesis of MS. Persistent and/or recurrent inflammatory processes are the main factor leading to the loss of neuroaxonal homeostasis and the onset of neurodegeneration that affects both white and gray matter [2].

This complex physiopathogenesis, with inflammatory and degenerative processes coexisting in different degrees and proportions, makes the optimization of the pharmacological therapy challenging, particularly for progressive forms of MS. Moreover, the presence of several symptoms such as spasticity, fatigue, ataxia and tremor, and bladder dysfunctions require a multidisciplinary approach and careful treatment selection.

The endocannabinoid system and related bioactive lipids participate in multiple physiological processes that include metabolic and immune regulatory mechanisms also contributing to the maintenance of the organism’s homeostasis [4]. Among endocannabinoid related bioactive lipids, N-acylethanolamines (NAEs) have similar chemical structures to endocannabinoids (ECs), but they do not show affinity for cannabinoid receptors. However, they do compete with ECs for metabolizing enzymes and thus can intensify the action of ECs by reducing their degradation (“entourage” effect) [5, 6]. The NAE functions are additionally mediated via different non-cannabinoid receptors and ion channels including various GPCRs (e.g., GPR55, GPR119), peroxisome proliferator-activated receptors (PPARs), and transient receptor potential (TRP) channels (e.g., vanilloid receptor TRPV1) [7–9]. Among NAEs, N-palmitoylethanolamine (palmitoylethanolamide, PEA) is abundant in the CNS and conspicuously produced by neurons and glial cells [10]. Evidence indicates that PEA is an important anti-inflammatory, analgesic, and neuroprotective mediator acting at several molecular targets in both central and peripheral nervous systems [9]. In addition to large evidence deriving from experimental studies, in recent years, several clinical studies have confirmed the antinflammatory and neuroprotective properties of PEA in humans [9, 11]. Most clinical trials with PEA were performed with formulations whose particles have been subjected to the so-called fluid jet micronization process. This allows to obtain particles with a defined size profile being completely different and statistically lower (6–10 μm at most) in comparison to naïve PEA (in the 100–700 μm range). Moreover, the ultramicronization process yields a different crystalline structure with higher energy content. The smaller particle size (with higher surface-to-volume ratio) combined with increased potential energy contributes to better solubility. These characteristics result in better diffusion and distribution of micronized and ultramicronized PEA compared to the naïve form, and thus superior biological efficacy [9, 12]. Among formulations containing ultramicronized PEA, PEALut also contains luteolin (3′,4′,5,7-tetrahydroxyflavone, Lut) [13].

Lut is a flavonoid present in many plants, including various fruits, vegetables, and medicinal herbs. Lut and its congeners exhibit antinflammatory, antioxidant, neuroprotective, and anti-carcinogenic activities and have been reported to potentially contribute to the treatment of MS [14].

In addition, Lut has been also shown to improve the morphology of PEA: naïve PEA shows a morphology characterized by large flat crystals; the coprecipitation of PEA with very small quantities of Lut stabilizes the microparticles inhibiting PEA crystallization process through creation of hydrogen bonds between PEA and Lut [15]. The greater molecular stability of PEA in the presence of Lut likely contributes to the high recovery of neurological outcome observed in experimental models of spinal cord injury, traumatic brain injury, and Alzheimer disease [16–18], as well as the clinical improvement observed in human stroke patients [19]. Furthermore, our previous findings showed that PEALut enhances oligodendrocyte progenitor cell (OPC) morphological complexity along with the expression of different myelin proteins [20, 21]. In OPCs subjected to tumor necrosis factor alpha (TNF-α) treatment, PEALut reduces the acute-phase protein serum amyloid A1 (SAA1) expression [22], a gene coding for SAA protein that was found to be elevated in peripheral blood of patients with relapsing–remitting MS over a 3-month period [23]. This raises the possibility that the composite might have protective effects in MS experimental models. To verify this hypothesis, the mouse model of experimental autoimmune encephalomyelitis (EAE) based on active immunization with a fragment of myelin oligodendrocyte glycoprotein (MOG_35-55_) was used. The daily assessment of clinical score and the expression of SAA1, proinfiammatory cytokines TNF-α, IL-1β, and IFN-γ, and NLRP3 inflammasome as well as TLR2, Fpr2, CD3-γ, TCR- ζ chain, CD137, and cannabinoid receptors CB_1_, CB_2_, and myelin basic protein (MBP) were evaluated in the brainstem and cerebellum at different postimmunization times (postimmunization days, PIDs).

## Methods

### EAE model and clinical neurological scoring

EAE model and clinical neurological scoring were performed as previously reported [24]. Animals were habituated for at least 1 week before the experiment and then were immunized. The animals were housed under controlled environmental conditions (LD 12:12 h regime in air-conditioned rooms, 22 ± 2 °C, 55 ± 10% humidity) and allowed free access to food and water throughout the experiment. To minimize stress factors and to accustom the animals to the experimenter, during the week preceding immunization, mice were gently handled by the same experimenter on alternate days (1 min per mouse/day). The number of animals for each experimental group and for each analyzed time was 8, if not otherwise specified.

All experiments were conducted in compliance with EU guidelines for the care and use of laboratory animals and those of the Italian Ministry of Health (D.Lg. 26/2014). The study was approved by the Institutional Review Board for Animal Research (Organismo Preposto al Benessere Animale, OPBA) of the University of Padua and by the Italian Ministry of Health (autorization number 65/2017-PR).

#### Induction of EAE and monitoring of the course of the disease

For induction of EAE, Hooke Kits™ EAE Emulsion (Hooke Laboratiores, Lawrence, Massachusetts USA) were used. C57BL/6 mice were immunized by administration of MOG_35–55_/Freund’s complete adjuvant (FCA) emulsion. Emulsion (200 μl) was administered subcutaneously at two sites (100 μl between the ears and 100 μl in hind flank). Each mouse was also injected intraperitoneally with 100 μl of Bordetella pertussis toxin (PTx) dissolved in phosphate-buffered saline (PBS) (Hooke Laboratiores) 4 h after the administration of the emulsion and again 24 h later. PTx enhances EAE development by providing additional adjuvant and is believed to facilitate entrance of autoimmune T cells into the CNS. Control mice were injected with CFA and PTx only (Hooke Control Kits™, Hooke Laboratiores, USA). All mice were weighed weekly and examined daily for the neurological symptoms of EAE, scored as reported in Table 1.

**Table 1:** EAE neurological scores

Score	Clinical signs
0	No sign of disease; the tail is erect, and locomotor activity is intact
0.5	Tail is erect, but the tip is limp
1.0	Tail is limp but signs of movement remain. Locomotor activity is slightly impaired and hind legs are extended
1.5	Tail is limp and one of two hind legs appears to be weak. Balance is slightly impaired
2.0	Tail is limp with no signs of movement; both hind legs are weak. Locomotor activity is impaired, and mice show poor balance and may drag a hind leg. Even in the absence of uncoordinated movement, the mouse presents with inclined head and poor balance
2.5	Tail is limp, both hind legs are weak; one hind leg lacks movement or may be dragging
3.0	Tail is limp and both hind limbs are completely paralyzed. Locomotor activity is clearly impaired
3.5	Tail is limp and both hind limbs are completely paralyzed, partial front leg paralysis. Locomotor activity is clearly impaired. When turned on its side, mouse is unable to right itself
4.0	Tail is limp, complete hind leg, and partial front leg paralysis. In cases where the mouse reaches a score 4 for 2 consecutive days, euthanasia is recommended
4.5	Complete hind and partial front leg paralysis, no movement around the cage. Mouse is not alert. Euthanasia is recommended
5.0	Mouse is dead

#### Compound treatment

PEALut for treatment was prepared as a 9 mg/ml stock solution in aqueous 10% (*w*/*v*) Pluronic F-68 and sonicated for 20 min in a sonicating water bath. The PEALut suspension was then diluted into PBS to reach 0.5 mg/ml (final concentration of Pluronic F-68: 0.55%). Mice received intraperitoneally 200 μl of either 0.55% Pluronic F-68 or 0.5 mg/ml PEALut to achieve a dose of 5 mg/kg body weight (assuming an average weight of 20 g per mouse). For lower doses, the PEALut stock solution was diluted accordingly in 0.55% Pluronic F-68, in order to maintain constant the concentration of carrier administered. PEALut was administered on a daily basis and at the same time each day. Clinical score was determined as described above.

For the dose response study, PEALut was administered at 0.1, 1, and 5 mg/kg starting from 11 PID until 27 PID. Mice (8 for each group) were sacrificed on 28 PID. For quantification of mRNAs, the dose of 5 mg/kg PEALut was employed and mice (6 for each group) were sacrificed at 7, 14, and 21 PIDs. All the mRNA expressions were evaluated on mice that received PEALut starting at 11 PID.

In a separate experiment, PEALut (5 mg/kg) treatment was started at 2 PID (8 for each group). Control animals were treated with PeaLut vehicle (Veh).

#### Tissue collection and processing

Animals of different experimental groups were sacrificed by cervical dislocation. Animals used for the evaluation of mRNAs were sacrificed before the onset of clinical score (7 PID), during the increase of clinical score (14 PID), and when the disease progression was ceased (21 PID). The brainstem and cerebellum were rapidly dissected out and suddenly frozen on dry ice. Tissues were stored at − 80 °C until processed for RNA extraction.

### Quantification of mRNAs related to inflammatory markers and to other proteins involved in MOG_35-55_-induced clinical alterations.

#### Quantitative real-time polymerase chain reaction (q-PCR)

Total RNA was extracted from brainstem and cerebellum by QIAzol (Quiagen), according to the manufacturer’s instructions. RNA quantity and purity were determined by NanoDrop 2000 (Thermo Scientific) (A260/280 ratio > 1.8). Reverse transcription was performed with Superscript IV reverse transcriptase (Thermo Scientific). The q-PCR reaction was performed as described previously [22]. Primer sequences are listed in Table 2. Amounts of each gene product were calculated using linear regression analysis from standard curves, demonstrating amplification efficiencies ranging from 90 to 100%. Dissociation curves were generated for each primer pair, showing single product amplification. Data are normalized to GAPDH mRNA level.

**Table 2:** Primer sequences used for reverse transcription

Gene	Forward (5′ → 3′)	Reverse (5′ → 3′)
GAPDH	TGGTGAAGGTCGGTGTGAAC	AATGAAGGGGTCGTTGATGG
SAA1	ACACTGACATGAAGGAAGCTAAC	GACCCCTTTGAGCAGCATCAT
TNF-α	CAAGTGGAGGAGCAGCTGGA	CATCGGCTGGCACCACTAGT
IL-1β	CTGGTGTGTGACGTTCCCATTA	CCGACAGCACGAGGCTTT
IFN-γ	ACATGAAAATCCTGCAGAGCCA	TCAGGTGTGATTCAATGACGCT
NLRP3	CCTGACCCAAACCCACCAGT	AGACCTCCCCAATGTGCTCG
TLR2	GCGGTCACTGGCAGGAGA	CATCTTTTCCACTTCTAGGTCGC
Fpr2	TCCTGGGCTCAAACTGATGA	GCAACAATTGACATGGGCAT
CD137	TCTGTGCTTAAGACCGGGACC	ATGGTGGTACTGGGAGAGAAGC
CD3γ	AGTGGCTTAAAGACGGGAGC	CCTCGAGGGTCTTTGGCATTG
TCR ζ chain	GAGCTTTGGTCTGCTGGATCCC	ACAGGGCTGTGATGATGACTCC
CB_1_	GATCTTAGACGGCCTTGCAGA	AATGTCATTTGAGCCCACGTAG
CB_2_	ACAGCTCCAGTAGAAGAAGCCA	TGAACTCCAAGCCACCGTTG
MBP	TCCGAGGAGAGTGTGGGTTT	TGGAACGATCTGGAGGGTTT

### Statistical analysis

Data are given as mean ± SEM at each time point. Statistical analyses to determine group differences were performed by ANOVA (Prism 7.0, Graphpad software) followed by Bonferroni multiple comparison test or Newman-Keuls for comparisons involving more than two data groups.

## Results

### PEALut treatment ameliorates clinical severity of EAE mice

**Fig. 1:**
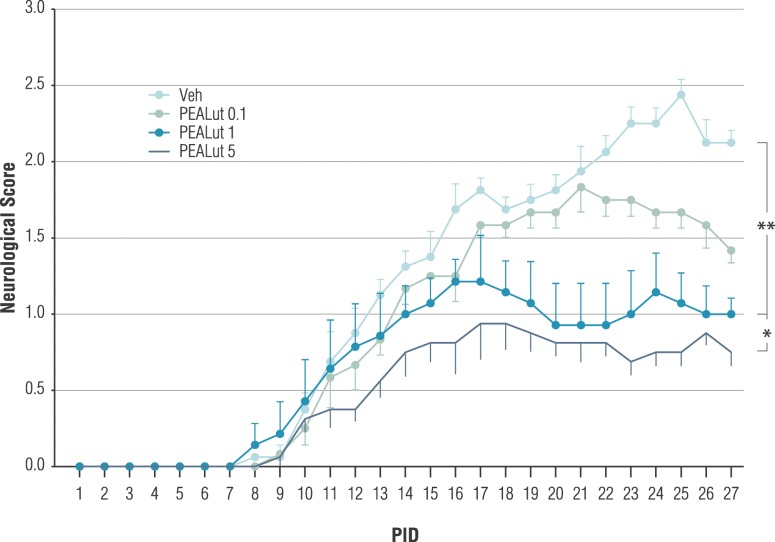
Dose-dependent effect of PEALut, started at 11 PID, on neurologic score severity in MOG_35-55_ immunized mice. All mice were immunized with MOG_35-55_ and treated with PEALut at doses of 0.1, 1, and 5 mg/kg or corresponding Veh. PEALut treatments started at 11 PID. Data are presented as means ± SD. *n* = 8 for each experimental group. *p = 0.04; **p = 0.004. Newman-Keuls post hoc test

**Fig. 2:**
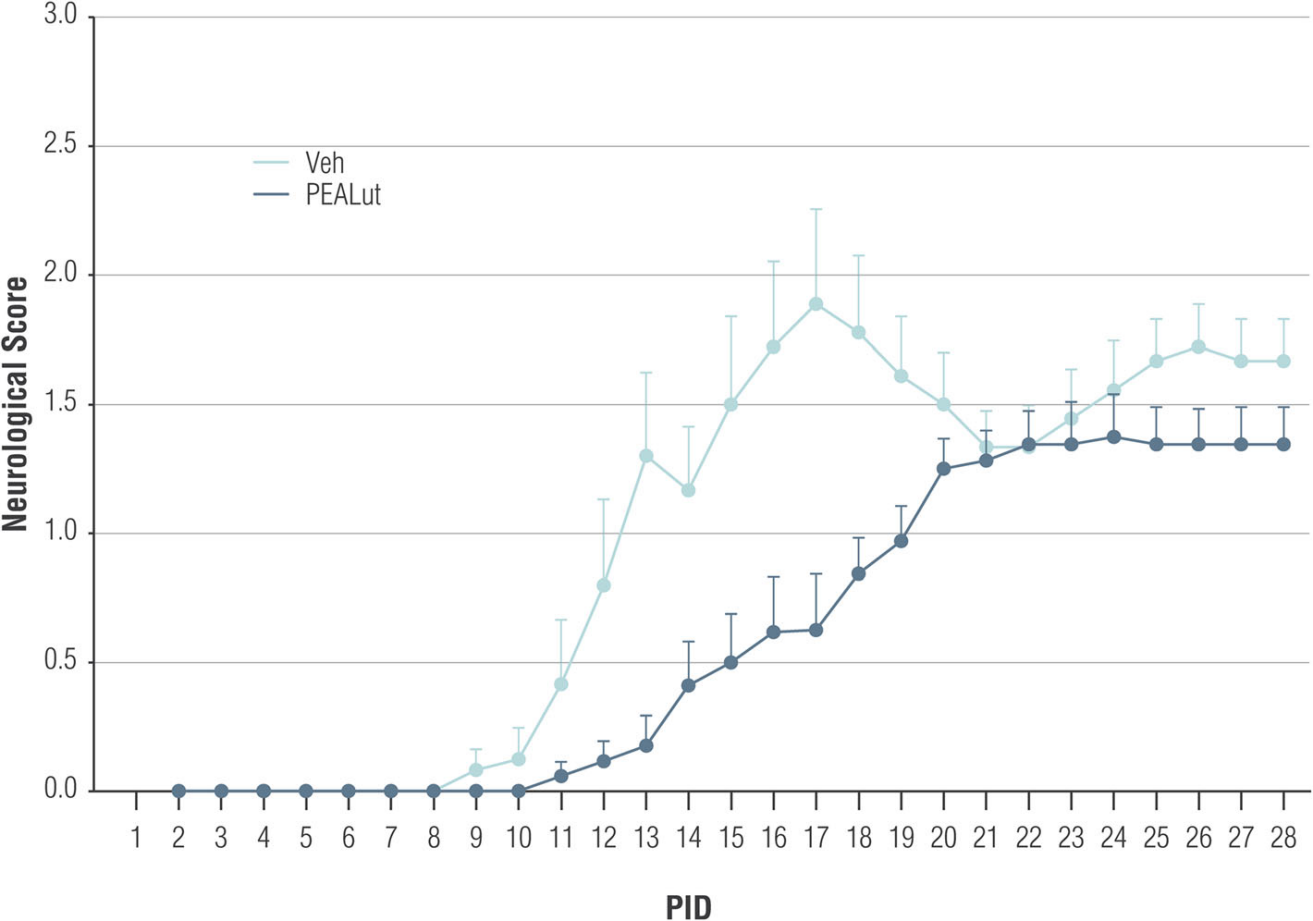
Effect of PEALut treatment, started at 2 PID, on neurologic score severity in MOG_35-55_ immunized mice. All mice were immunized with MOG_35-55_ and treated with PEALut at 5 mg/kg or corresponding Veh starting at 2 PID. Data are presented as means ± SD. *n* = 8 for each experimental group. The PEALut treatment induced a significant reduction in score severity (*p* = 0.04, repeated measure ANOVA)

All groups of mice started to develop clinical signs, including tail and hindlimb paralysis, on days 8–9 after MOG_35-55_ immunization. Veh-treated mice developed a typical course of chronic EAE, as evidenced by ascending paralysis which peaked several days later and then persisted until the end of the experiment (Figs. 1 and 2). The treatment with PEALut to MOG_35-55_ mice starting at 11 PID elicited a significant reduction in clinical symptoms (*p* = 0.002; repeated measure ANOVA). Furthermore, the PEALut induced a dose-dependent reduction in clinical score. In fact, the treatment with 0.1 mg/kg failed to affect EAE course, while 1 and 5 mg/kg significantly attenuated the paralysis of EAE during the observation period as compared to the Veh treated group (*p* = 0.022 and *p* = 0.004, respectively, Newman-Keuls post hoc test; Fig. 1). The reduction in clinical scores elicited by 5 mg/kg PEALut was significantly higher as compared to 1 mg/kg (*p* = 0.04). A significant PEALut-induced reduction in neurological scores, without a complete inhibition of the immune response induced by MOG_35-55_, was also observed when PEALut treatment (5 mg/kg) started at 2 PID (*p* = 0.04, repeated measure ANOVA, Fig. 2).

### PEALut treatment starting at 11 PID reduces the inflammatory response after MOG_35-55_ peptide immunization

The mRNA expression of the acute-phase SAA1, proinflammatory cytokines TNF-α, IL1-β, and IFN-γ, and NLRP3 inflammasome were evaluated in the brainstem and cerebellum at 7, 14, and 21 PIDs. Non-immunized mice were used as control.

**Fig. 3:**
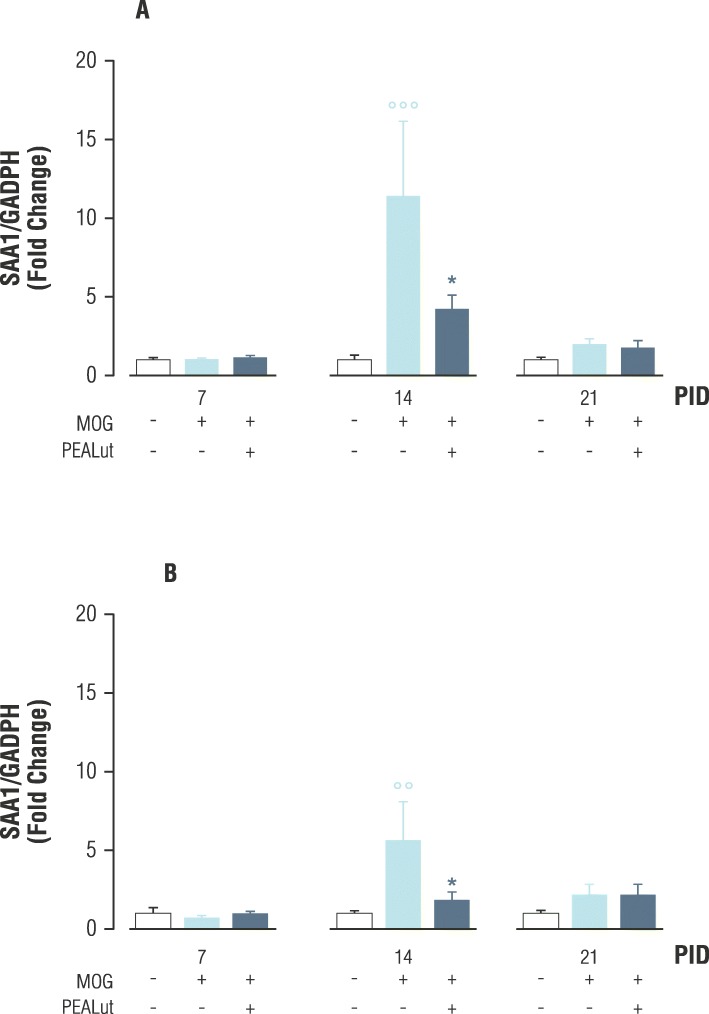
SAA1 mRNA expression in MOG_35-55_-mice treated with Veh or PEALut. Mice were treated with Veh, MOG/Veh, or MOG/PEALut for 7, 14, and 21 days. Veh or PEALut treatment started at 11 PID. SAA1 mRNA values are reported as mean ± SEM. **a** Brainstem, **b** cerebellum. Data are shown as relative expression of Veh values normalized to 1. *n* = 6 mice for each group. °°*p* < 0.01, °°°*p* < 0.001 compared with the Veh-treated group. *p < 0.05 compared with the MOG/Veh

SAA1 mRNA level was low in control mice in both the brainstem and cerebellum at all analyzed times (Fig. 3). In Veh-MOG_35-55_ mice, a significant increase of SAA1 mRNA, as compared to non-immunized controls, was observed in both the brainstem and cerebellum at 14 PID (*p* < 0.001 and *p* < 0.01, respectively. Bonferroni post hoc test; Fig. 3) but not at 21 PID. In MOG_35-55_ mice treated with 5 mg/kg PEALut, the increase of SAA1 mRNA observed at 14 PID was statistically downregulated as compared to Veh-MOG35-55 mice (*p* < 0.05; Bonferroni post hoc test; Fig. 3).

TNF-α, IL-1β, and IFN-γ transcripts were expressed at low level in both the brainstem and cerebellum in Veh-treated animals. A significant mRNA upregulation of all analyzed cytokines was observed in Veh-MOG_35-55_ mice at 14 PID, but not at 7 and 21 PID, in both the brainstem and cerebellum (*p* < 0.001 for TNF-α and IFN-γ; *p* < 0.05 for IL-1β; Bonferroni post hoc test). In MOG/PEALut-treated mice, the increase in TNF-α, IL-1β, and IFN-γ mRNAs was significantly reduced as compared to MOG_35-55_ mice receiving (*p* < 0.05; Bonferroni post hoc test, Table 3).

**Table 3:** Proinflammatory protein mRNAs expression in brainstem and cerebellum of MOG_35-55_ immunized mice treated with Veh or PEALut

		Brainstem			Cerebellum		
		PID			PID		
		7	14	21	7	14	21
TNF-α/GADPH	Veh	1.0 ± 0.1	1.0 ± 0.3	1.0 ± 0.2	1.0 ± 0.2	1.0 ± 0.1	1.0 ± 0.2
	MOG/Veh	2.5 ± 0.3	106.4 ± 33.3°°°	21.6 ± 6.5	1.6 ± 0.1	71.7 ± 19.7°°°	32.2 ± 14.1
	MOG/PEALut	2.4 ± 0.4	52.6 ± 21.9*	15.9 ± 4.5	2.0 ± 0.2	31.9 ± 11.4**	16.5 ± 4.6
IL1-β/GADPH	Veh	1.0 ± 0.1	1.0 ± 0.2	1.0 ± 0.2	1.0 ± 0.1	1.0 ± 0.5	1.0 ± 0.5
	MOG/Veh	2.4 ± 0.6	330.2 ± 77.8°°°	31.5 ± 6.9	2.1 ± 0.3	123.0 ± 78.6°	21.8 ± 8.8
	MOG/PEALut	4.7 ± 2.0	174.7 ± 65.8**	23.7 ± 6.5	4.0 ± 0.8	18.8 ± 12.2*	14.8 ± 6.8
INFγ/GADPH	Veh	1.0 ± 0.2	1.0 ± 0.5	1.0 ± 0.4	1.0 ± 0.2	1.0 ± 0.4	1.0 ± 0.2
	MOG/Veh	1.0 ± 0.3	115.2 ± 0.5°°°	19.2 ± 4.7	1.7 ± 0.5	37.8 ± 15.7°°	27.8 ± 8.1
	MOG/PEALut	1.4 ± 0.3	35.7 ± 17.2**	19.3 ± 7.7	1.0 ± 0.1	6.9 ± 1.8*	22.5 ± 10.4
NLRP3/GADPH	Veh	1.0 ± 0.1	1.0 ± 0.1	1.0 ± 0.2	1.0 ± 0.1	1.0 ± 0.1	1.0 ± 0.1
	MOG/Veh	0.7 ± 0.0	11.2 ± 1.4°°°	3.3 ± 0.6	0.7 ± 0.1	7.2 ± 2.0°°°	4.4 ± 0.8°
	MOG/PEALut	1.0 ± 0.2	6.9 ± 2.2**	2.2 ± 0.6	0.9 ± 0.1	2.9 ± 0.6**	2.9 ± 0.6

Mice were treated with Veh, MOG/Veh, or MOG/PEALut for 7, 14, and 21 days. Veh or PEALut treatments started at 11 PID. TNF-α, IL-1β, INFγ, and NLRP3 mRNAs values are reported as mean ± SEM. Data are shown as relative expression of Veh-treated values normalized to 1. *n* = 6 mice for each group. °*p* < 0.05, °°*p* < 0.01, and °°°*p* < 0.001 compared with the Veh-treated group. *p < 0.05 and **p < 0.01 compared with the MOG/Veh

The expression of NLRP3 was markedly upregulated in MOG_35-55_ immunized-mice at 14 PID (*p* < 0.001; Bonferroni post hoc test), and such increase was still significant at 21 PID in the cerebellum (Table 3). In MOG/PEALut-treated mice, there was a downregulation of NLRP3 mRNA in the cerebellum at 14 PID.

### MOG_35-55_ peptide and PEALut treatment regulate the expression of selected receptors involved in inflammation in EAE mice

The expression of TLR2, Fpr2, CD3-γ, TCR-ζ chain, CD137, CB_1_, CB_2_, and MBP mRNAs were evaluated in the brainstem and cerebellum at 7, 14, and 21 PIDs. In the former area, TLR2, Fpr2, CD3-γ, TCR-ζ chain, CD137, and CB_2_ mRNAs showed a significant upregulation in Veh-MOG_35-55_ mice at 14 PID, whereas only CD3-γ, TCR-ζ chain, and CB_2_ were still upregulated at 21 PID. In the cerebellum, TLR2, Fpr2, CD3-γ, TCR-ζ chain, CD137, and CB_2_ mRNAs were upregulated at 14 PID and the expression of TLR2, CD3-γ, TCR-ζ chain, and CB_2_ mRNAs persisted at 21 PID (Table 4). CB_1_ and MBP transcripts have not changed in expression at any time. In MOG/PEALut-treated mice, CD3-γ, TCR-ζ chain, CD137, TLR2, and CB_2_ mRNAs were significantly downregulated as compared to Veh-MOG_35-55_ mice at 14 PID in both brain areas (Table 4).

**Table 4:** Expression of TLR2, Fpr2, CD3-γ, TCR-ζ chain, CD137, CB_1_, CB_2_, and MBP mRNAs in the brainstem and cerebellum of MOG_35-55_ immunized mice treated with Veh or PEALut

		Brainstem			Cerebellum		
		PID			PID		
		7	14	21	7	14	21
TLR2/GADPH	Veh	1.0 ± 0.2	1.0 ± 0.2	1.0 ± 0.1	1.0 ± 0.1	1.0 ± 0.4	1.0 ± 0.3
	MOG/Veh	0.7 ± 0.2	12.0 ± 2.8°°°	4.6 ± 0.8	0.7 ± 0.0	17.3 ± 7.6°°	16.2 ± 6.6°
	MOG/PEALut	1.0 ± 0.3	6.8 ± 1.9*	3.2 ± 0.6	0.8 ± 0.4	2.6 ± 0.7*	9.7 ± 4.8
Fpr2/GADPH	Veh	1.0 ± 0.2	1.0 ± 0.1	1.0 ± 0.1	1.0 ± 0.4	1.0 ± 0.2	1.0 ± 0.1
	MOG/Veh	1.4 ± 0.5	45.6 ± 11.8°°°	8.0 ± 2.8	1.3 ± 0.3	50.7 ± 26.2°°	32.3 ± 13.0
	MOG/PEALut	1.6 ± 0.5	24.6 ± 9.1*	5.1 ± 1.4	1.9 ± 0.2	22.6 ± 11.2	17.3 ± 4.1
CD3γ/GADPH	CTRL	1.0 ± 0.1	1.0 ± 0.1	1.0 ± 0.1	1.0 ± 0.2	1.0 ± 0.1	1.0 ± 0.1
	MOG/Veh	1.2 ± 0.6	72.7 ± 9.1°°°	44.6 ± 11.4°°°	1.5 ± 0.4	140.4 ± 72.2°°	151.0 ± 40.7°°
	MOG/PEALut	1.3 ± 0.3	35.2 ± 14.4**	31.8 ± 8.8	0.9 ± 0.3	31.0 ± 13.8*	105.7 ± 33.9
TCR-ζchain/GADPH	CTRL	1.0 ± 0.3	1.0 ± 0.1	1.0 ± 0.1	1.0 ± 0.4	1.0 ± 0.1	1.0 ± 0.1
	MOG/Veh	0.9 ± 0.2	45.1 ± 8.0°°°	19.3 ± 4.6°°	1.4 ± 0.3	10.6 ± 4.5°°	8.5 ± 1.9°
	MOG/PEALut	0.8 ± 0.2	19.2 ± 7.5***	13.1 ± 3.3	1.4 ± 0.2	3.2 ± 1.1*	7.1 ± 2.2
CD137/GADPH	Veh	1.0 ± 0.2	1.0 ± 0.1	1.0 ± 0.2	1.0 ± 0.2	1.0 ± 0.1	1.0 ± 0.1
	MOG/Veh	1.0 ± 0.0	140.7 ± 47.0°°°	11.9 ± 2.6	1.4 ± 0.0	27.9 ± 13.4°°°	7.3 ± 1.4
	MOG/PEALut	1.2 ± 0.0	50.9 ± 20.3**	9.6 ± 2.2	1.8 ± 0.4	5.9 ± 1.2**	6.0 ± 1.4
CB_2_/GADPH	Veh	1.0 ± 0.2	1.0 ± 0.2	1.0 ± 0.3	1.0 ± 0.0	1.0 ± 0.3	1.0 ± 0.2
	MOG/Veh	1.2 ± 0.1	19.0 ± 5.7°°°	7.4 ± 2.0°°	0.9 ± 0.1	8.1 ± 2.8°°°	7.5 ± 1.9°°
	MOG/PEALut	1.5 ± 0.1	6.5 ± 2.5**	6.3 ± 2.3	1.5 ± 0.3	2.9 ± 0.5*	5.7 ± 1.8
CB_1_/GADPH	Veh	1.0 ± 0.2	1.0 ± 0.3	1.0 ± 0.2	1.0 ± 0.5	1.0 ± 0.2	1.0 ± 0.1
	MOG/Veh	0.8 ± 0.2	1.1 ± 0.3	1.2 ± 0.3	1.0 ± 0.2	0.9 ± 0.2	1.1 ± 0.1
	MOG/PEALut	0.8 ± 0.1	1.3 ± 0.5	1.1 ± 0.3	1.6 ± 0.4	1.1 ± 0.3	0.9 ± 0.1
MBP/GADPH	CTRL	1.0 ± 0.2	1.0 ± 0.1	1.0 ± 0.1	1.0 ± 0.0	1.0 ± 0.1	1.0 ± 0.1
	MOG/Veh	1.1 ± 0.0	0.9 ± 0.0	0.8 ± 0.0	0.8 ± 0.1	0.8 ± 0.2	1.1 ± 0.1
	MOG/PEALut	1.1 ± 0.1	1.0 ± 0.1	1.0 ± 01	1.0 ± 0.1	0.8 ± 0.1	0.9 ± 0.0

Mice were treated with Veh, MOG/Veh, or MOG/PEALut for 7, 14, and 21 days. Veh or PEALut treatments started at 11 PID. TLR2, Fpr2, CD3γ, TCR-ζ chain, CD137, CB_1_, CB_2_, and MBP mRNA values are reported as mean ± SEM. *n* = 6 mice for each group. Data are shown as relative expression of Veh-treated values normalized to 1. *n* = 6 mice for each group. °*p* < 0.05, °°*p* < 0.01, and °°°*p* < 0.001 compared with the Veh-treated group. *p < 0.05, **p < 0.01, and ***p < 0.001 compared with the MOG/Veh

## Discussion

The present results demonstrate that the intraperitoneally administration of the composite PEALut significantly reduces the development of clinical signs in the MOG_35-55_ model of EAE. The dose-dependent improvement of clinical score induced by PEALut is associated with a reduction in transcript expression of the acute-phase protein SAA1, the pro-inflammatory cytokines TNF-α, IL-1β, IFN-γ, and NLRP3, and of receptors TLR2, Fpr2, CD137, CD3-γ, TCR-ζ chain, and CB_2_.

For dose-response evaluation, PEALut administration was initiated at 11 PID, at the first signs of sickness. At this time, the T cell priming is not prevented, and it is possible to assess just the subsequent decrease of the disease (semi-therapeutic treatment). A protective effect was also observed when the PEALut treatment was started at 2 PID. In this condition, PEALut attenuates the clinical symptoms induced by MOG_35-55_. This suggest that the reduced clinical symptoms could be due to a reduced T cell infiltration into the CNS also indicated by CD3-γ and TCR-ζ chain mRNA reduction in the PEALut-treated group.

In a previous study [25], it was demonstrated that 5 mg/kg PEA reduced the severity of clinical scores of EAE. In the present paper, using the same EAE protocol, but administering different doses of a composite containing PEA, PEALut, we obtained a reduction in neurological scores that resembles what was previously reported [25]. The results of our study confirm the anti-inflammatory effects of PEALut, which resembles to that of PEA alone. In contrast, in our in vitro studies, PEALut regulation of gene expression in oligodendrocyte precursor cells was not mimicked by its molecular components alone, thus suggesting a putative novel effect of PEALut on myelination processes [20]. Unfortunately, in our in vivo experimental conditions, in both the brainstem and cerebellum, we did not observe any change in gene codifying myelin proteins at any time (Table 4). This did not allow the evaluation of the PEALut effect on myelination. To test a potential PEALut remyelinating effect, it should be more convenient to use the classical and well established cuprizone-mediated model of demyelination/remyelination.

In addition to neurological scores, in the present study, we examined gene expression and receptors involved in inflammation in the brainstem and cerebellum. Furthermore, we followed the development of EAE over the time analyzing a large number of inflammatory gene markers (such as, TNF-α, IL1-β, INF-γ, SAA1, and NLRP3) and the expression of selected receptors involved in inflammation (such as TLR2, Fpr2, CD3-γ,TCR-ζ chain, and CB_2_).

In Rahimi’s paper [25], the TNF-α, IFN-β, and IL-17 gene expression was examined only at 28 PID; in their experimental conditions, these genes were still upregulated in MOG_35-55_-treated animals. In our conditions, the gene expression was evaluated at 7, 14, and 21 PID. At 14 PID, the expression of all inflammation-related genes was remarkably increased in both the brainstem and cerebellum and their expression was significantly reduced by PEALut. At 21 PID, there was no longer upregulation of inflammatory gene markers induced by MOG_35-55_-immunization; in fact, all the genes examined were not significantly different from the control animals.

PEALut has been shown to act as protective agent in different experimental models of CNS diseases [16, 19, 26]. Its activity has been shown to be superior to ultramicronized-PEA [17]. The active molecules of composite, PEA and Lut, have complementary and additive pharmacological activities, due to the ability to interact with different targets involved in the inflammatory response [27, 28]. PEA is endowed with important neuroprotective, anti-inflammatory, and analgesic actions. The peroxisome proliferator-activated receptor (PPAR)-α is the molecular target that directly mediates some of the neuroprotective, anti-inflammatory, and analgesic effects of PEA [6]. Indirect mechanisms of action for PEA has also demonstrated that PEA potentiates anandamide actions at cannabinoid receptors (while itself having no appreciable affinity for either CB_1_ or CB_2_ receptors) and anandamide desensitization of transient receptor potential cation channel subfamily V member 1 (TRPV1) channels (“entourage” effects). Lut is a widespread flavone known to have antioxidant and cytoprotective properties related to nuclear factor erythroid 2-related factor 2-(Nrf2) pathway. Extensive in vitro and in vivo investigations have underlined that Lut exhibits beneficial neuroprotective properties via different mechanisms [28]. Importantly, Lut has been shown to improve the morphology of PEA: naïve PEA shows a morphology characterized by large flat crystals, and the presence of very small quantities of Lut stabilizes the microparticles inhibiting PEA crystallization process [15]. The stabilization of PEA by the addition of Lut together with the combination of two different active molecules make PEALut an interesting multi-target product with high potentiality to tackle the chronic complex condition affecting CNS. The reduction in clinical signs in EAE MOG_35-55_ model alongside the decrease of the expression of different transcripts involved in the neuroinflammation suggests that PEALut might have protective effects in MS, as observed in other CNS diseases.

To identify possible mechanisms underlying PEALut-induced protection in EAE mice, we evaluated the expression of genes coding for inflammatory proteins, i.e., SAA1, TNF-α, IL-1β, IFN-γ, and NLRP3. PEALut has been shown to regulate inflammatory processes by modulating the production of cytokines in different CNS conditions associated with neuroinflammation alongside a modulation of NF-κB activation [17, 26], and the present data extend the effect to a condition that summarize many aspects of MS. Moreover, this is the first evidence concerning the regulation of NLRP3 expression by a NAE derivative in CNS neuroinflammation. The NLRP3 has been reported to be involved in the development of MS through the secretion of IL-1β and IL-18 [29], and its pharmacological inhibition has been regarded as a potential target for the treatment of MS [30]. Enhanced expression of NLRP3 is known to occur in response to NF-κB activation [31]. Since PEALut has been reported to limit NF-κB activation [17, 26], the PEALut-induced NLRP3 mRNA decrease might be a consequence of the modulation of NF-κB activation. Further studies are necessary to establish a potential direct action of PEALut on NLRP3 expression.

In our study, PEALut also elicited a reduced SAA1 expression. In our knowledge, this is the first evidence concerning the ability of the composite PEALut to regulate SAA1 mRNA expression in two cerebral areas involved in EAE development. Interestingly, PEALut has been found to significantly limit the rise of SAA1 mRNA expression in oligodendrocyte progenitor cells subjected to a 1-week exposure to TNF-α [22]. SAA1 is an acute phase protein, known to be synthesized mainly by hepatocytes in response to cytokines and other regulatory factors. These proteins mobilize the leukocyte population to neutralize pathogens, while simultaneously initiate the repair processes to restore normal function and limit secondary inflammatory damage [32].

SAA protein was found to be elevated in peripheral blood of patients with relapsing–remitting MS over a 3-month period [23]. In chronic-relapsing (CR) EAE models of MS, the contribution of the response in the acute phase was recently reported [33]. In CR-EAE, SAA1 mRNA was found to be significantly elevated in the liver in mice before the onset of clinical signs. In the present study, by using the EAE MOG_35-55_ model, we find that SAA1 mRNA is also significantly increased in the brainstem and cerebellum in the symptomatic phase of the disease. Since SAA1 mRNA was not measured immediately before the symptomatic phase, it is not clear whether its production preceded the onset of neurological signs as observed in the liver of CR-EAE mice. SAA1 mRNA transcription is constitutive in human and mouse brain [34–36]. Pyramidal neurons of the cerebral cortex and Purkinje cells of the cerebellum have been reported to express SAA1 mRNA in normal human brain [36]. However, non-neuronal SAA1 mRNA expression occurs, in CNS, in glial cell populations, and its synthesis is stimulated by TNF-α, lipopolysaccaride, and exogenous recombinant human Apo-SAA [22]. This evidence suggests that the increased SAA1 mRNA in the brain areas of EAE mice could be produced by activated glial cells, including oligodendrocytes. In line with this hypothesis, in the brains of patients with neurologically confirmed MS, an intense immunohistochemical staining of SAA is associated to the myelin sheaths of axons in the affected areas [37]. Moreover, PEALut has been shown to reduce SAA1 mRNA in OPCs after TNF-α treatment [22]. However, the possibility of a neuronal increase or a synthesis of SAA1 mRNA by immune cells recruited from periphery cannot be ruled out.

Many pro- and anti-inflammatory signals and pro-resolving circuits converge on a group of receptors that integrate contrasting cues to determine the course of inflammation. Among these receptors, we report that the mRNA of receptors CD137, CD3-γ, CTR-ζ chain, TLR2, Fpr2, and CB_2_ was increased in the brain areas of MOG_35-55_, mainly on 14 PID, when clinical symptoms were well established. Their expression was regulated by PEALut administration. All these receptors are known to be linked, with specific functions, to inflammatory response; however, little is known about their temporal profiles in the brainstem and cerebellum of mice immunized with MOG_35-55_ peptide. The costimulatory molecule CD137 (4-1BB, TNFRSF9), a member of the TNF receptor superfamily, is critical for both the induction and effector phase of EAE. CD137 and CD137 ligands (CD137L, 4-1BB ligand, TNFS9) increase in serum and CSF of MS patients [38]. In addition, mRNA CD137 has been reported to increase in activated memory TMOG cells and its transcription regulated by non-psychoactive cannabinoid, cannabidiol [39]. The reduction in CD137 mRNA expression, induced by PEALut, might be also related to activated T cells. The data sustain the idea that manipulation of costimulatory signals might represent an important mechanism to inhibit immune activation.

The possibility of PEALut to influence T cell activation and/or infiltration is also suggested from its ability to reduce the expression of the CD3-γ and TCR-ζ chain, two transcripts of the TCR signal-transduction complex. PEALut effects on T cell markers suggest that less T cells infiltrate the CNS, and therefore the autoimmune response may have been inhibited at peripheral sites rather than a central sites.

As far as TLR2 evaluation in EAE models are concerned, a previous in situ hybritization study reported a positive staining of TLR2, mainly across meninges, on 10 PID as well as a robust expression in cerebral parenchima in several brain regions, including the medulla and cerebellum, 3 weeks after the MOG_35-55_ immunization, when animals showed severe clinical symptoms [40]. Our results confirm the increase of TLR2 mRNA expression in connection with clinical symptom presence; nevertheless, the highest expression was observed on 14 PID in both the brainstem and cerebellum. Since dual labeling performed in previous study [40] provided the anatomical evidence that microglia/macrophages were the cells expressing TLR2 in the brain of EAE mice, the decrease in TLR2 mRNA elicited by PEALut confirms that microglia are one of TLR2 targets to reduce neuroinflammation. A further receptor expressed by immune cells is the Fpr2. We report, for the first time, a marked increase of Fpr2 mRNA in the brainstem and cerebellum of mice immunized with MOG_35-55_ on 14 PID and an important reduction elicited by PEALut treatment. Fpr2 is a G protein-coupled receptor functionally expressed on immune cells, including mast cells, microglia, and epithelial cells, that transduces signals from lipoxin A4, annexin A1, and SAA to regulate inflammation and its resolution [41, 42]. Fpr2 ligand-specific interactions are able to induce either proinflammatory or proresolution/anti-inflammatory effects. In conditions associated to CNS damage such as the hemicerebellectomy, Fpr2 protein level was significant upregulated in secondary inflammatory damage phase [43]. Our results show that an upregulation in Fpr2 also occurs in experimental models of MS and that its regulation with PEALut might trigger the resolution of MOG_35-55_-induced inflammation. The finding that PEALut also limits MOG_35-55_-induced increase of CB_2_ mRNA also argue for a promotion of inflammation resolution induced by the composite. In CNS, the expression of CB_2_ receptors is mainly related to the activation state of microglia and upregulation of CB_2_ receptors has been associated with a restoration of tissue homeostasis in pathological neuroinflammatory conditions [44]. On the other hand, CB_2_ agonists include endogenous bioactive lipids such as endocannabinoids that are well characterized pro-resolving lipid mediators [45]. Importantly, the ability of PEA to enhance endocannabinoid tone is well known [46].The data of this report suggest that PEALut might also enhance endocannabinoid tone. In contrast to CB_2_ gene expression, we did not find any change in the CB_1_ receptor gene expression. In our knowledge, this is the first time that the CB_1_ gene is evaluated in MOG_35-55_-EAE model. Previous reports showed a decrease in CB_1_ receptors in the monophasic model of EAE in rats that was restricted to the basal ganglia and cortical structures [47]. Thereafter, a moderate decrease in the density of CB_1_ receptors was described in the caudate–putamen during the acute phase of chronic relapsing EAE while a more marked extent in the chronic phase [48]. This evidence shows that the alteration of CB_1_ are region-dependent and become more pronounced during the chronic phase of the disease. Our results are in line with previous studies: in fact, the MOG_35-55_ immunization condition used results in an acute monophasic EAE, with a moderate severity of neurological score.

A limitation of the present study relates and concerns the lack of information on the protein levels corresponding to the upregulated genes. In our previous study, PEALut was found to stimulate both the MBP gene expression alongside with the MBP [20]. Unfortunately, in the EAE model employed, in both the brainstem and cerebellum, we did not observe any change in genes codifying myelin proteins at any time (data not shown). For this reason, we could not investigate the in vivo effect of PEALut on gene expression and protein levels of myelin proteins, as we had done in in vitro studies [20].

In the present study, we also scheduled to measure TNF-α protein with ELISA in the brainstem at 28 PID. At this time, unlike to what we had thought, both gene expression and protein levels return to basal levels in both MOG-Veh and PEALut-treated groups.

## Conclusions

The results reported here demonstrate that PEALut reduced the severity of clinical signs in the MOG_35-55_ model of EAE throughout a multitude of anti-inflammatory signals and pro-resolving circuits. The results reported do not clarify whether PEALut might also be useful to reduce the demyelination associated with MS; in fact, the experimental conditions adopted and the methods used did not allow the observation of demyelinating processes. The use of demyelination models independently of immune attack such as the cuprizone demyelination model will be more suitable to verify whether PEALut facilitates the development of OPCs in vivo and modulate the expression of different myelin proteins as observed in vitro [20, 21].

## Data Availability

The datasets generated and/or analyzed during the current study are available from the corresponding author on reasonable request.

## Abbreviations

CB_1_: Cannabinoid receptor type 1
CB_2_: Cannabinoid receptor type 2
CD137: Tumor necrosis factor receptor superfamily member 9
CD3-γ: T cell co-receptor CD3 γ chain
CNS: Central nervous system
EAE: Experimental autoimmune encephalomyelitis
EC: Endocannabinoid
FCA: Freund’s Complete Adjuvant
Fpr2: N-formyl peptide receptor 2
GAPDH: Glyceraldehyde-3-phosphate dehydrogenase
GPCR: G protein-coupled receptor
IFN-γ: Interferon gamma
IL-1β: Interleukin 1 beta
Lut: Luteolin
MBP: Myelin basic protein
MOG_35-55_: Fragment of myelin oligodendrocyte glycoprotein
MS: Multiple sclerosis
NAE: N-Acylethanolamine
NLRP3: NLR pyrin domain containing 3 inflammasome
OPC: Oligodendrocyte progenitor cell
PEA: Palmitoylethanolamide
PEALut: Co-ultramicronized PEA and Lut
PID: Post-immunization day
PPAR: Peroxisome proliferator-activated receptors
PTx: Bordatella pertussis pertussis toxin
SAA1: Serum amyloid A1
TCR-ζ chain: T cell surface glycoprotein ζ chain
TLR2: Toll-like receptor 2
TNF-α: Tumor necrosis factor alpha
TRP: Transient receptor potential channels
